# Supplementary material for: Genome-wide identification, characterization and gene expression of BES1 transcription factor family in grapevine (Vitis vinifera L.)
Source: Sci Rep. 2023 Jan 5;13:240. doi: 10.1038/s41598-022-24407-y (PMC9816167; doi:10.1038/s41598-022-24407-y)
Supplement: Supplementary file 3 — Supplementary Information. [file 41598_2022_24407_MOESM3_ESM.zip › Vvi_Atr/Vitis_vinifera.PN40024.v4.dna_sm.toplevel.fa.vs.Amborella_trichopoda.AMTR1.0.dna_sm.toplevel.fa.html/Atr-AmTr_v1.0_scaffold00176.html]

|  |  |  |  |  |  |  |  |  |  |  |  |  |  |
| --- | --- | --- | --- | --- | --- | --- | --- | --- | --- | --- | --- | --- | --- |
| Duplication depth | Reference chromosome | Collinear blocks | | | | | | | | | | | |
| 0 | Atr-ERN11361 |  |  |  |  |  |  |  |  |  |  |  |  |
| 0 | Atr-ERN11362 |  |  |  |  |  |  |  |  |  |  |  |  |
| 0 | Atr-ERN11363 |  |  |  |  |  |  |  |  |  |  |  |  |
| 0 | Atr-ERN11364 |  |  |  |  |  |  |  |  |  |  |  |  |
| 1 | Atr-ERN11365 |  | Vvi-Vitvi01g00512\_t001 |  |  |  |  |  |  |  |  |  |  |
| 1 | Atr-ERN11366 |  | \| \| |  |  |  |  |  |  |  |  |  |  |
| 1 | Atr-ERN11367 |  | \| \| |  |  |  |  |  |  |  |  |  |  |
| 1 | Atr-ERN11368 |  | \| \| |  |  |  |  |  |  |  |  |  |  |
| 1 | Atr-ERN11369 |  | \| \| |  |  |  |  |  |  |  |  |  |  |
| 1 | Atr-ERN11370 |  | Vvi-Vitvi01g00509\_t001 |  |  |  |  |  |  |  |  |  |  |
| 1 | Atr-ERN11371 |  | Vvi-Vitvi01g00508\_t001 |  |  |  |  |  |  |  |  |  |  |
| 1 | Atr-ERN11372 |  | \| \| |  |  |  |  |  |  |  |  |  |  |
| 1 | Atr-ERN11373 |  | \| \| |  |  |  |  |  |  |  |  |  |  |
| 1 | Atr-ERN11374 |  | \| \| |  |  |  |  |  |  |  |  |  |  |
| 1 | Atr-ERN11375 |  | Vvi-Vitvi01g00503\_t002 |  |  |  |  |  |  |  |  |  |  |
| 1 | Atr-ERN11376 |  | \| \| |  |  |  |  |  |  |  |  |  |  |
| 1 | Atr-ERN11377 |  | Vvi-Vitvi01g00501\_t001 |  |  |  |  |  |  |  |  |  |  |
| 1 | Atr-ERN11378 |  | \| \| |  |  |  |  |  |  |  |  |  |  |
| 1 | Atr-ERN11379 |  | \| \| |  |  |  |  |  |  |  |  |  |  |
| 1 | Atr-ERN11380 |  | Vvi-Vitvi01g01995\_t001 |  |  |  |  |  |  |  |  |  |  |
| 1 | Atr-ERN11381 |  | Vvi-Vitvi01g00500\_t001 |  |  |  |  |  |  |  |  |  |  |
| 1 | Atr-ERN11382 |  | \| \| |  |  |  |  |  |  |  |  |  |  |
| 1 | Atr-ERN11383 |  | \| \| |  |  |  |  |  |  |  |  |  |  |
| 1 | Atr-ERN11384 |  | Vvi-Vitvi01g00499\_t001 |  |  |  |  |  |  |  |  |  |  |
| 1 | Atr-ERN11385 |  | \| \| |  |  |  |  |  |  |  |  |  |  |
| 1 | Atr-ERN11386 |  | Vvi-Vitvi01g00498\_t001 |  |  |  |  |  |  |  |  |  |  |
| 0 | Atr-ERN11387 |  |  |  |  |  |  |  |  |  |  |  |  |
| 0 | Atr-ERN11388 |  |  |  |  |  |  |  |  |  |  |  |  |
| 0 | Atr-ERN11389 |  |  |  |  |  |  |  |  |  |  |  |  |
| 0 | Atr-ERN11390 |  |  |  |  |  |  |  |  |  |  |  |  |
| 0 | Atr-ERN11391 |  |  |  |  |  |  |  |  |  |  |  |  |
| 0 | Atr-ERN11392 |  |  |  |  |  |  |  |  |  |  |  |  |
| 0 | Atr-ERN11393 |  |  |  |  |  |  |  |  |  |  |  |  |
